# Synthetic Biodegradable Microparticle and Nanoparticle Vaccines against the Respiratory Syncytial Virus

**DOI:** 10.3390/vaccines4040045

**Published:** 2016-12-02

**Authors:** Patricia A. Jorquera, Ralph A. Tripp

**Affiliations:** Department of Infectious Disease, College of Veterinary Medicine, 111 Carlton Street, University of Georgia, Athens, GA 30602, USA; jorquera@uga.edu

**Keywords:** vaccine, nanoparticle, microparticle, RSV, VLPs

## Abstract

Synthetic biodegradable microparticle and nanoparticle platform technology provides the opportunity to design particles varying in composition, size, shape and surface properties for application in vaccine development. The use of particle vaccine formulations allows improvement of antigen stability and immunogenicity while allowing targeted delivery and slow release. This technology has been design to develop novel vaccines against the respiratory syncytial virus (RSV), the leading cause of lower respiratory tract infection in infants. In the last decade, several nano- and micro-sized RSV vaccine candidates have been developed and tested in animal models showing promising results. This review provides an overview of recent advances in prophylactic particle vaccines for RSV and the multiple factors that can affect vaccine efficacy.

## 1. Introduction

The airways are the major point of entry for many pathogens. Among them, viruses, such as the respiratory syncytial virus (RSV), influenza (flu) A and B, parainfluenza (PIV) 1, 2 and 3 and adenovirus (AdV), are capable of penetrating the initial defenses of the upper respiratory tract (UTR), infect the lower respiratory tract (LRT) and cause a great burden in terms of severe disease in humans [1]. RSV causes significant morbidity and mortality in children younger than five years of age and is a major public health burden worldwide. It infects more than 90% of children at least once before the age of two, accounting for approximately 70% of hospitalizations due to bronchiolitis [2,3,4]. In the elderly, RSV infection often results in obstructive pulmonary disease as a complication due to underlying pulmonary and cardiac disease [5]. Unfortunately, RSV infection does not confer long-term protection causing reinfections throughout life, which creates a significant disease risk in individuals with cardiopulmonary disease, immunocompromised patients and the elderly [5]. No safe and effective RSV vaccine is available, and thus, prevention of RSV infection is limited to standard infection control practices.

Vaccines are a key component in the control and prevention of respiratory infections. Vaccination is an affordable way to control viral diseases. Selecting the right vaccine antigen to induce a protective and long-lasting immune response can be difficult, especially when the antigen is a poor immunogen. However, a successful vaccine may be achieved by using a combination adjuvant and delivery system, such as synthetic biodegradable particles carriers. These particles have adjuvant properties that can enhance and direct the immune response to the antigen by different mechanism that depend on the type of particle and/or co-delivered adjuvant [6,7]. Vaccine adjuvants can be broadly classified into two groups based on their modes of action: (1) those that work as immune potentiators; and (2) those that work as delivery systems that promote antigen uptake [7]. The adjuvant effect of particle carriers has been reported for many years. Particle carriers include: nanoparticles (NPs), microparticles (MPs), virosomes [8], virus-like particles (VLPs) [9,10,11,12], emulsions, Immune stimulating complexes (ISCOMs) [13,14] and liposomes [15], which have all been shown to work effectively. This has been attributed to the comparable dimensions to pathogens, which they emulate, and how they are presented to the immune system. Some other ways that these carriers work are: (1) serving as an effective antigen delivery system that enhances antigen uptake by antigen-presenting cells (APCs); (2) increasing the availability of antigens to APCs by working as a depot for controlled release of antigen; (3) modulating the type of immune response induced when used alone or in combination with immunostimulatory compounds; (4) protecting the integrity of the antigen by preventing its degradation; and/or (5) promoting antigen cross-presentation to CD8+ T cells.

This short review provides an overview of the next generation of nanoparticle and MP-based RSV vaccines. To understand how vaccination could prevent RSV transmission and lower respiratory tract infection, there is a need to understand the hurdles associated with the infection of the respiratory mucosa and induction of adaptive immune response.

## 2. Lymphoid Tissue and Immune Response in the Respiratory Tract

**Figure 1:**
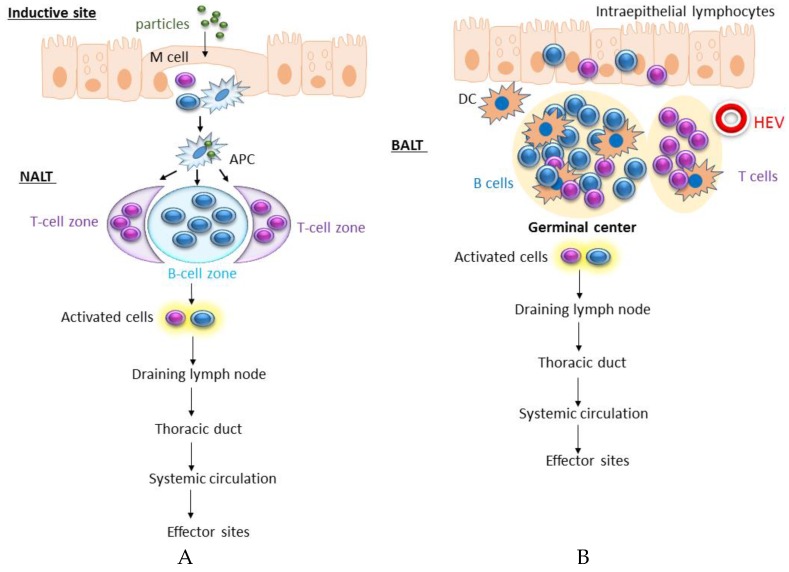
The nasal-associated lymphoid tissue (NALT) and bronchus-associated lymphoid tissue (BALT). (**A**) The NALT contains many lymphoid follicles (B cell areas), interfollicular areas (T cell areas) and antigen-presenting cells (APCs, macrophages and dendritic cells). The epithelium forming the NALT comprises ciliated epithelial cells, goblet cells and non-ciliated cells similar to the M-cells. Activated T and B cell are transported to the draining lymph nodes (dLNs) by efferent lymphatics draining and from the dLNs to the effector sites; (**B**) BALT is less organized than NALT and is located mostly at bifurcations of the bronchial tree. BALT is a structure with T cell and B cell areas, high endothelial venules (HEV) in the T cell zone and an overlying lymphoepithelium containing cells similar to the microfold cells (M). It is composed of undifferentiated macrophages and lymphocytes in a loose stromal network, with an epithelium lacking of the goblet cells and cilia.

In humans, the lymphoid tissues constituting the upper respiratory tract are the adenoids, bilateral tubule and palatine and lingual tonsils (Waldeyer’s ring) [16]. The existence of a nasal analog of the gut-associated lymphoid tissue (GALT) or bronchus-associated lymphoid tissue (BALT) remains unclear. An equivalent of these secondary lymphoid tissue aggregates termed nasal-associated lymphoid tissues (NALT) has been described in rodents [17]. In mice, efferent lymphatics drain from NALT into the cervical lymph nodes of the upper thorax, but no afferent lymphatics are present. NALT also contains aggregates of lymphoid follicles (B cell areas), interfollicular areas (T cell areas), macrophages and dendritic cells (DCs) (Figure 1A).

The presence of BALT varies between species, being common in rabbits and rats and mostly absent in healthy humans and mice [18]. BALT is less organized than NALT and is located mostly at bifurcations of the bronchial tree [17]. BALT is a structure with T cell and B cell areas, high endothelial venules in the T cell zone and an overlying lymphoepithelium containing cells similar to the microfold (M) cells of the Peyer′s patches (Figure 1B). It is composed of undifferentiated macrophages and lymphocytes in a loose stromal network, with an epithelium lacking the goblet cells and cilia. The lymphocytes are often enfolded by M cells to form clusters [19]. These M cells have the ability to selectively phagocyte and pinocyte particles and molecules of the lumen of the airway [19]. Efferent lymphatics drain to the regional lymph nodes, but no afferent lymphatics are seen leading to BALT follicles [17]. T cell areas are found localized in a parafollicular distribution, and the majority of cells within BALT are B cells expressing surface IgA and IgM, but plasma cells are found only around the periphery of BALT [17]. The normal immune responses occur for both inhaled particles and infectious agents, and this takes place primarily in the draining mediastinal lymph nodes and not directly in BALT [20,21]. Infectious agents, chronic inflammation and/or autoimmune responses can induce BALT formation, and these structures are termed inducible BALT (iBALT) [18,22]. iBALT is different from the classical BALT in that it does not always possess an overlying epithelium; it may form in the lung parenchyma and is not always found associated with the airway [18].

RSV infection takes place initially in the ciliated columnar cells of the URT, and then, it spreads to the LRT largely by aspiration of virus-containing secretion into the trachea and by direct cell-to-cell spread [23]. RSV inhibits cilial beat and induces loss of cilia from the epithelial surface [16]. Studies in calves infected with bovine RSV have shown that RSV does not replicate in regional lymph nodes or palatine tonsils, although virus-positive cells can be found in the epithelial cells covering the pharyngeal tonsil [23]. At Day 4 post-infection, the infection spreads to the bronchiolar and alveolar epithelium, perhaps as a result of the cilia destruction of the bronchial epithelium. In the alveoli, RSV antigen can be detected in both type I and type II pneumocytes; however, type II pneumocytes are the main site of RSV replication [23]. Neutrophils seem to be the main phagocytizing cell of RSV antigens and particles that reach the bronchial lumen [23]. Alveolar macrophages (AM) are important in clearing neutrophils and debris [23]. It is believed that AM are non-migratory and do not contribute to the induction of adaptive immune responses; however, it has been shown that in mice, AM can migrate from the lung to the draining lymph node (dLN) and transport bacteria to this site, although their contribution to the induction of adaptive immune response in the dLN remains unknown [24]. The number of pulmonary macrophages can increase substantially in response to virus infection as a result of an influx of blood monocyte precursors and from local cell proliferation, and they may serve as APCs for activated effector T cells in the infected lungs [25].

Pulmonary dendritic cells reside below the airway epithelial cells within the pulmonary interstitium. DCs can extend processes between the epithelial cells making them capable of sampling viral particles in the inflamed lung and migrate to the dLN in a CCR7-dependent manner [24]. During this process, DCs upregulate the expression of co-stimulatory and adhesion molecules (such as CD40, CD80, CD86 and ICAM1) and MHC molecules, rendering them competent to serve as potent APCs for the activation of naive T cells in the dLNs [26]. Naive and memory virus-specific T cells go through a stepwise process of activation, proliferation and differentiation to become effector T cells that can migrate to the site of infection and mediate antiviral immune responses [26] or they can become RSV-specific T follicular helper (Tfh) cells that can support the differentiation of extrafollicular B cells into plasma cells. Naive B cells are located in the LNs and in the lung interstitium. Viral infection results in the formation of tertiary lymphoid structures (iBALT) along the branching points of the bronchial tree [22]. iBALT contains B cell areas, germinal centers and antibody-forming cells (AFC). Binding of the antigen (Ag) induces a complex set of events that ultimately results in the formation of both Ag-specific memory B cells and plasma cells. Plasma cells are effector B cells that secrete large amounts of antibodies (Abs), no longer express MHC class II or respond to T cell help and do not proliferate [27]. Exposure of B cells to T-independent antigens induces the proliferation and formation of foci of AFC that produce and secrete Abs with relatively low affinity for the Ag [27]. AFC can be detected as early as three days post-infection in the cervical and mediastinal LNs, and in the lung, AFC produce mainly IgG and IgM [28]. Exposure of B cells to T-dependent antigens results in the activation and establishment of a germinal center (GC). Germinal center B cells, in association with DCs and Tfh cells, undergo rapid proliferation, somatic hypermutation and affinity maturation [27]. When B cells exit the GC, they become: memory B cells that circulate in the blood, splenic marginal zone B cells [29] or plasma cells that migrate to the bone marrow and secrete high-affinity Abs [27].

In mice, RSV infection elicits the generation of substantial numbers of RSV-specific plasma cells in the bone marrow that produce antibodies directed against the F and G proteins; however, in the NALT, RSV-specific plasma cell numbers wane quickly both after primary and secondary infection [30]. This indicates that the inability to generate a robust local mucosal response in the nasal tissues may contribute to the likelihood of subsequent reinfection and that the presence of serum anti-RSV antibody without local protection is not enough to protect against reinfection. In addition, it has been shown that the RSV G protein CX3C chemokine motif can modulate the immune response, resulting in a reduced Th1- and increased Th2-type T cell phenotype, as well as reduction in pulmonary recruitment of cytotoxic T cells expressing CX3CR1 [31]. Given the nature of RSV and the fact that reinfections in the presence of antibodies are common through life, it is unlikely that a live-attenuated vaccine will induce sterilizing immunity. A more realistic goal would be to develop a vaccine that promotes T cell response and long-lasting antibody response to prevent the development of severe RSV disease, linked to the infection of the lower respiratory tract. An effective vaccine may not completely prevent virus infection, but it may perhaps limit RSV replication to the upper respiratory tract and prevent viral spreading to the deeper airways, resulting in a reduction of hospitalization rates. The use of NPs and/or MP vaccines to ensure efficient antigen delivery to APCs and proper T and B cell activation may have a role here.

## 3. The Effect of the Particle Size

Generally speaking, nanoparticles are defined as solid particles ranging in size from 10 to <1000 nm [16], and MPs as particles ranging in size from 1 to <1000 µm [32]. The particles are made of biodegradable materials that allow the release of the antigen as the particle degrades over time. During its formulation, the antigen payload can be encapsulated, adsorbed, attached or captured into these particles, and the final combination of biodegradable material plus the antigen will ultimately determine the size and shape of the particle, as well as its immunogenicity.

**Figure 2:**
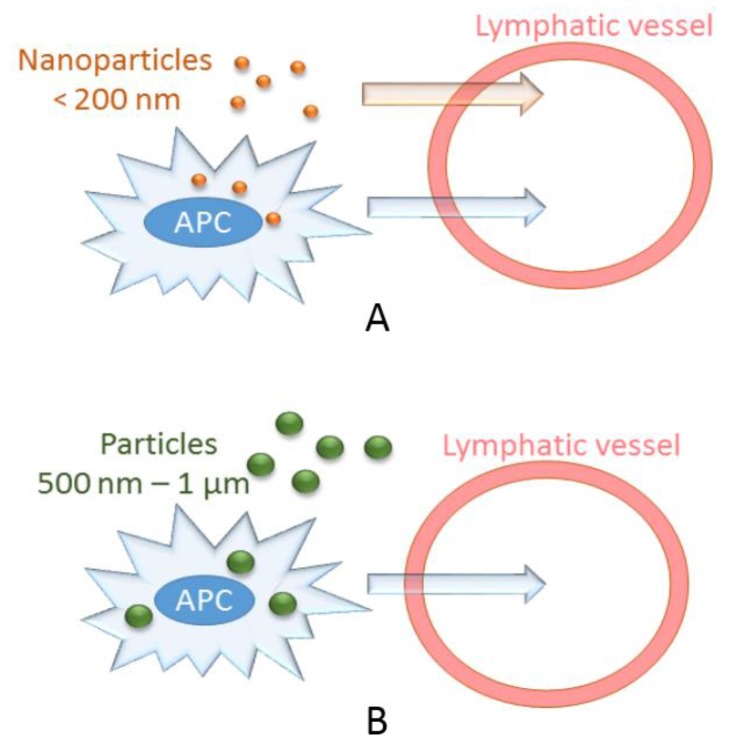
The size of the particle affects its entry into the initial lymphatic vessel. (**A**) Particles smaller than 200 nm in diameter can efficiently enter the lymphatic system either freely or are transported by antigen presenting cells (APCs); (**B**) Particles larger than 500 nm require active transport by APCs to the lymph nodes.

The route of delivery of these particles may also affect its immunogenicity. NPs and MPs need to reach secondary lymphoid organs to induce adaptive immune responses, and this traffic to the draining lymph node occurs in a size-dependent manner [33]. The lymphatic vessels can transport particles and cells of up to several µm, depending on the diameter of the lymphatic vessel. Lymphatic vessels vary in size with initial vessels being 10–60 µm in diameter and larger vessels up to 2 mm in diameter [33]. Several features can influence the entry of particles into the initial lymphatic vessels, and size is an important factor. Molecules of 20–200 nm efficiently enter the lymphatic system, with particles smaller than 50 nm being more efficiently transported than those larger than 100 nm [33] (Figure 2). By contrast, particles larger than 200–500 nm do not efficiently enter lymph capillaries in a free from, and they require active transport by dendritic cells to the dLNs [33,34]. Particles 500 nm–2 µm in diameter are mostly associated with DCs from the injection site, whereas NPs (20–200 nm) are found in the lymph node-resident DCs and macrophages, suggesting free drainage of these particles to the LNs [35]. Similarly, the lymphatic uptake of liposomal particles upon subcutaneous injection is more efficient for small liposomes (40 nm) than for large liposomes (>400 nm); however, small liposomes are less efficiently retained in the dLN than larger liposomes; this is due to the more efficient phagocytosis of larger liposomes by macrophages [15].

**Figure 3:**
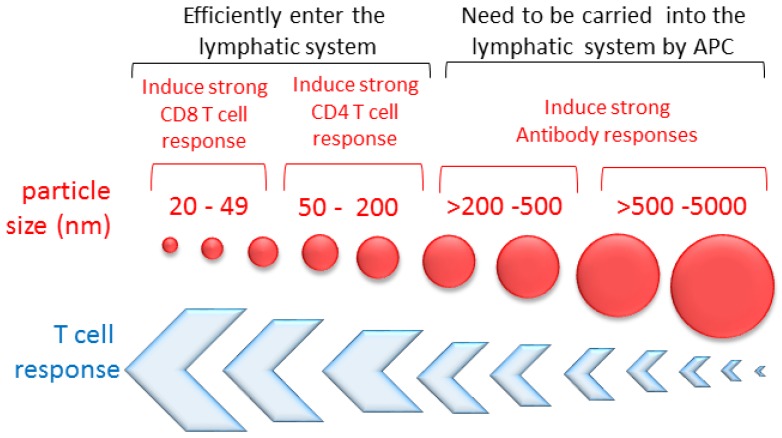
Effect of the particle size on the immune response. Particles 20–200 nm in diameter efficiently enter the lymphatic system, with particles smaller than 50 nm being more efficiently transported than those larger than 100 nm. Particles smaller than 49 nm in diameter induce a stronger T CD8 cell response, and particles larger than 50 nm promote a stronger CD4 T cell response. Overall, the strength of the T cell response inversely correlates with the particle size and antibody response, which seems to be stronger with larger particles.

A recent study assessed the effect of particle sizes ranging from 80–250 nm on the immunogenicity of oil-in-water emulsion adjuvants combined with RSV F protein. The authors showed that upon vaccination, the humoral and T cell response inversely correlated with the particle size, with the 80 nm particle size emulsion being the most immunogenic among the particles [36]. Other studies have shown that NPs smaller than 200 nm are more effective than MPs (>1 µm) at priming CD8 T cells [37], whereas MPs tend to induce stronger humoral response [38] (Figure 3).

Poly(d,l-lactic acid) (PDLLA) MPs administrated orally are efficiently taken up by Peyer′s patches (PP). MPs smaller than 5 µm in diameter are transported to the spleen, where the released antigen stimulates serum IgG response, whereas larger MPs (>5 µm) remain in the PP and stimulate IgA production [39,40] (Figure 3). Particle shape is also an important parameter that can affect its immunogenicity. The particle shape has been shown to affect the distribution of NPs following intravenous (i.v.) injection [41]. Short-rod NPs (~100 nm long) are easily trapped in the liver, while long-rod NPs (~800 nm long) distribute in the spleen [41]. Similarly, i.v. injection of filomicelles ~8 μm long persist for more time in circulation, but filomicelles that exceed ~8 μm undergo rapid fragmentation [42]. Under fluid flow conditions, spheres and short filomicelles are taken up by cells more readily than longer filaments because the latter are extended by the flow [42].

It has been shown that the particle size may favor a particular type of immunity, with small NPs (similar in size to viruses) inducing Th1-type responses and larger particles inducing Th2-type responses [16]. For example, NPs carrying ovalbumin (OVA) induced CD8 T cells’ response when the particle size was limited to 40–49 nm, while CD4 T cell activation and IL-4 were induced by particles 93–123 nm in diameter [37]. Since Th1-type responses are preferred for a robust response, while IL-4 is reported to cause asthma-like symptoms, smaller particles may be preferable for RSV vaccine delivery and targeting.

## 4. The Effect of Antigen Presenting Cells

Particulate vaccines are endocytosed by phagocytic cells, i.e., macrophages and dendritic cells. In general, particles of 50–80 nm are endocytosed through caveolae-mediated endocytosis [43,44,45]; particles <150 nm in diameter are taken up via classic receptor-mediated endocytosis through clathrin-coated pits [45,46]; and larger particles (>500 nm) are endocytosed via macropinocytosis and phagocytosis and restricted to professional antigen presenting cells (APC), such as macrophages and immature DCs [38]. Particles can also be endocytosed via clathrin- and caveolae-independent lipid raft-mediated mechanisms, but the size-restrictions are not well understood [44,46]. Upon internalization, the particles are hydrolyzed in the endosome/lysosomes of APCs, and antigens are then processed and complexed with MHC class II molecules for subsequent presentation and activation of CD4 T cells [47,48]. A fraction of the particles may escape from the endosome/lysosome into the cytoplasm causing the slow release of the antigen, its degradation and loading onto MHC class I molecules, resulting in the presentation and activation of CD8 T cells [47,49,50,51]. It is not known what contributes to the level of antigen cross-presentation; however, it has been shown that mannose receptor-mediated endocytosis promotes cross-presentation [52]. However, the pH of the endosomal compartment and the type of phagocytic cell (CD8^+^ DC or CD103^+^ DC) determine the course of events that lead to antigen cross-presentation [53,54].

It has been shown that dendritic cells can internalize particles as large as 15 μm in diameter in vitro, although particles in the size range of 1–15 μm are only phagocytosed by a minority of the dendritic cells [55]. Particles ≤500 nm in diameter are optimal for cell uptake, but the uptake of larger particles can be enhanced by rendering the particle surface positive [55,56]. Macrophages can also uptake PLGA MPs (~1 µm in diameter), and mannan and chitosan surface modification enhance endocytosis in a receptor-mediated manner [57].

## 5. The Route of Administration

The route of vaccine administration can significantly affect the immune response to the antigen. The parenteral and mucosal routes of administration are the most traditional approaches for licensed vaccines; however, novel ways to deliver vaccines have been explored in the last few years.

From the parenteral routes, intramuscular (i.m.) administration is one of the most common, mainly because the muscle has better vascularization than subcutaneous tissue, and serious reactions are far less common than during subcutaneous (s.c.) injection [58]. On the other hand, the s.c. route allows one to inject larger volumes than the intradermal (i.d.) route, and it may be used when a large amount of vaccine antigens needs to be injected. The i.d. route has become an attractive route of injection for particle antigens, because it has the potential of inducing strong immune response by targeting APCs located in the dermis (i.e., Langerhans cells).

RSV is a respiratory pathogen, and a defect in RSV-specific IgA response is linked to the susceptibility of infection in humans [59]. Vaccination that promotes IgA secretion in the respiratory mucosa, such as intranasal immunization, could potentially provide protection against RSV infection and transmission. The intranasal route of vaccination targets mainly the M cells and APCs in the NALT epithelium [60]. Murine NALT is quite different from that in humans; however, studies in mice have provided insight into the immunology of the human respiratory tract. Some of the advantages of i.n. vaccination is the non-invasive, needle-free administration and induction of mucosal and systemic immunity; however, the main disadvantage of this route is the rapid clearance and insufficient uptake of the antigen [60]. Particle vaccines composed of mucoadhesive polymers have the potential for enhancing absorption and retention of the antigen to facilitate its uptake by APCs. Polymers such as poly(lactic-co-glycolic acid) (PLGA), chitosan, alginate and carbopol have been previously explored [61]. PLGA nanoparticles carrying bovine PIV3 antigens have been shown to be efficient at eliciting mucosal IgA and serum IgG responses; however, cell-mediated immunity was not detected upon vaccination [62]. A recent study compared the effect of the incorporation of mannan (MN) and chitosan (CS) on PLGA MPs carrying the hepatitis B surface antigen. Intranasal vaccination with MPs carrying both MN and CS induced stronger humoral and cell-mediated immune responses than PLGA, MN-PLGA and CS-PLGA MPs, suggesting that incorporation of these two polymers may promote vaccine efficacy [57]. Intranasal vaccination of C57BL/6 mice with 61.5 nm-sized nanocomplexes of the RSV G protein (aa region 131–230) and polysorbitol transporter (PST) elicited mucosal and humoral antibody responses that were sustained for over 200 days [63]. These complexes were phagocytosed by macrophages, but they were also incorporated by A549 cells, suggesting that these osmotically-active complexes can be taken up by phagocytosis-independent mechanisms.

Buccal and sublingual vaccine deliveries have become an attractive route of mucosal vaccination because their anatomy and physiology have some advantages over other mucosal routes, such as a mucosal epithelium composed of stratified squamous, non-keratinized cells and the presence of Langerhans cells in the mucosa and myeloid DCs along the lamina propria [64]. For buccal and sublingual vaccination, the antigens can pass through the epithelium and are taken up by Langerhans cells and DCs, processed and presented to T and B cells. Recently, a salmonella-based RSV vaccine modified to carry RSV F and G epitopes was delivered orally to BALB/c mice; the results showed the induction of mucosal, humoral and cellular immunity upon vaccination, suggesting that this candidate could prevent RSV infection [65]. Sublingual administration of an adenoviral vector expressing the RSV F protein (HDAd-sFsyn) induced systemic and mucosal immune response in BALB/c mice with antibody titers sustained for up to 14 weeks after a single immunization that protected mice against RSV infection [66]. Comparison of intragastric, intranasal and intradermal delivery routes of plasmid DNA encoding RSV F protein encapsulated in PLGA MPs in BALB/c mice has been investigated. Intragastric and intranasal immunization induced very low levels of RSV-specific serum antibodies and did not provide protection against RSV [67]. In contrast, intradermal (i.d.) vaccination elicited strong humoral response, protected animals against RSV infection and promoted pulmonary IgG1 secretion [67]. Similarly, i.d. vaccination of mice with NPs (~25–50 nm) conjugated to CpG-B and CpG-C oligonucleotides in combination with NPs carrying OVA enhanced uptake of OVA-NPs by dendritic cells and promoted antigen cross-presentation, eliciting potent Th1 and CD8 T cell responses [68].

## 6. The Particle Material

The materials employed for the synthesis of NPs and MPs are derived from natural and synthetic sources. Polymeric particles carriers can be made from a variety of polymers, such as chitosan, alginate, silica, polylactide, polyethylenimine, propylene sulfide, poly (lactic-co-glycolic acid) (PLGA), polymethylmethacrylate (PMMA), poly(ethylacrylic acid) and poly(butyl acrylic acid) (reviewed in [69]). PLGA and chitosan are the two most widely used for vaccine delivery, and these two polymers have been recently evaluated on macrophages [70]. Chitosan MPs (3.6 µm in diameter) were cytotoxic to murine macrophages (J-774.1 cells), and PLGA MPs were innocuous; however, this study did not evaluate the cytotoxicity of these particles in vivo [70]. Chitosan is a biocompatible, biodegradable, nontoxic, cationic polysaccharide produced by the deacetylation of chitin. The cationic nature of chitosan allows it to form electrostatic complexes or multilayer structures with other negatively-charged synthetic or natural polymers [71]. Chitosan particles can be absorbed through the paracellular route on the epithelial layer, which is important for nasal or oral delivery of protein vaccines [71]. Chitosan NPs have been used to intranasally (i.n.) deliver RSV F peptides in fusogenic 6-helix bundle form and have been demonstrated to enhance pulmonary delivery of the peptide, as well as preventing the progression of pulmonary RSV infection [72]. A chitosan-encapsulated DNA-based RSV vaccine has been also tested [73]. Intranasal vaccination with a chitosan-DNA nanoparticle vaccine (MGXV) carrying a mixture of plasmid DNA encoding nine RSV antigens (NS1, NS2, M, SH, F, M2, N, G and P) induced serum IgG and mucosal IgA and CD8 T cell responses that protected mice against RSV infection and disease [73]. Another study evaluated DNA-chitosan NPs (80–150 nm) carrying the F, M2 and G genes and showed RSV protein expression in mouse tissues (liver, kidney, lung and heart) when the vaccine was injected via i.v.; however, vaccine efficacy was not tested in this study [74]. A more recent study used poly(2-hydroxyethyl methacrylate) (pHEMA) surrounded by a chitosan cationic shell to encapsulate an RSV F gene construct; the DNA vaccine was efficiently delivered in mouse skeletal muscle and resulted in high gene expression at the injection site [75]. Similarly, chitosan NPs have been used to deliver siRNA directed against RSV NS1, and this was shown to protect rats against RSV challenge. Since NS1 protein can block IFN expression, inhibiting NS1 synthesis can potentially balance the immune response toward Th1 and, therefore, improve viral clearance [76].

Alginate is an anionic unbranched biopolymer composed of guluronic acid and mannuronic acid residues. Due to its gelation and mucoadhesive properties, alginate nanoparticles can prolong the antigen release and increase the immune response [77]. Alginate has been used to coat chitosan NPs carrying measles antigen to improve vaccine stability when given orally [78]. Oral vaccination of BALB/c mice with alginate-coated chitosan NPs induced humoral and mucosal immune response against measles that inversely correlated with the molecular size of chitosan [78]. Importantly, this study provides evidence that alginate coating protected the NPs in the gastric environment, suggesting that alginate-coated chitosan NPs may be used for the oral delivery of therapeutics.

Poly(lactic-*co*-glycolic) acid, or PLGA, has gained considerable interest as a base material for vaccine formulation due to its clinically-proven biocompatibility, biodegradation, potential for interaction with other biological materials and approval for clinical use in humans by the FDA [79]. Biodegradable PLGA nano- and MPs have become popular due to their capacity to facilitate the uptake of encapsulated materials and to protect different molecules, such as peptides/protein antigens and nucleic acids, increasing delivery efficiency and promoting Ag uptake by APCs. PLGA MPs carrying a plasmid DNA encoding the RSV G protein have been tested [80]. G-PLGA MPs elicited an RSV-specific CD8 T response, promoted a balanced Th1/Th2 response and partially protected BALB/c mice against RSV challenge [80]. Similarly, intramuscular (i.m.) vaccination of RSV-experienced mice with PLGA MPs (14.8 µm) carrying the G2Na peptide (G protein aa region 130–230) proved to be efficacious and elicited antibody responses for at least 148 days. Importantly, vaccination with this formulation in the presence of anti-RSV antibodies did not compromise avidity maturation of the antibodies over time [81]. Calves vaccinated i.n. with PLGA MP vaccine carrying peptides of the bovine RSV F and G proteins elicited nasal IgA secretion, but not serum IgG or cellular response; however, vaccination reduced the overall incidence of respiratory disease in these animals [82].

Silica NPs have been used for vaccine delivery; however, this material is less common than PLGA and chitosan. Mesoporous silica NPs and MPs show good cellular uptake by human monocyte-derived DCs and low cytotoxicity [83]. Silica NPs have adjuvant-like properties and can enhance mucosal and systemic immune response in mice when given orally [84]. Recently, it was shown that cationic mesoporous silica nanoparticles (130 nm) loaded with ovalbumin can be formulated into sugar-based MPs to create nanoparticle-in-microparticle structures with a size range between 20 and 40 µm that could be potentially used for epidermal powder immunization (EPI) [85]. Since the sugar matrix composed of trehalose, mannitol and dextran is water soluble, these MPs could dissolve after EPI delivery into the skin.

A recent study used NP-forming thermo-responsive polymers (TRP) for co-delivery of RSV F protein trimers adjuvanted with toll-like receptor (TLR)-7 and TLR-8 agonists [86]. Amine conjugation of TLR-7/-8 agonists to the F trimers disrupted the recognition of neutralizing epitopes, whereas coupling of the F trimers to TRP nanoparticles retained the antigenicity and induced high titers of antibodies directed against the pre-fusion form of the F protein that protected against RSV challenge [86]. These results suggest that TRP NPs may provide a novel platform for eliciting neutralizing antibodies to structure-dependent epitopes in other pathogens.

Layer-by-layer (LbL) assembly of poly(amino acids) is another method through which multilayered films can be fabricated on substrates to create nano- and MPs. These particles can have a solid core (core-shell particles) or can be generated as hollow-shell particles (capsules) upon removal of the core [87]. LbL nanoparticles made of poly-l-lysine and poly-l-glutamic acid were loaded with the RSV G protein CX3C motif, which have been tested in mice and shown to promote cellular response and antibody response that protected them from RSV challenge [88]. Likewise, immunization with synthetic LbL microparticle vaccines carrying the G protein CX3C motif and the M2 CD8^+^ epitope, administered using a microneedle patch, elicited humoral and cellular immune responses and protected mice from challenge with RSV [89].

Virus-like particles (VLPs) are generated from viral proteins that self-assemble into structures resembling native viral particles and that have similar antigenicity of the native virus from which they were derived (reviewed in [9]). The size of the VLPs is similar to that of nanoparticles, ranging from 20 nm to 100 nm. Due to their size, they share some properties with NPs; for example, they can enter freely into the lymphatic vessel and reach the draining lymph nodes [9]. Several platforms for producing VLPs have been employed to develop RSV vaccines carrying the F, G and/or M2 proteins, such as insect cells [10,90,91], mammalian cells [11,92,93,94], plant expression system [95,96], bacteria [97,98] and in vitro cell-free systems [8,99]. A variety of VLP-based RSV vaccines has been developed and tested in the last decade. All of them have been shown to provide either total or partial protection against RSV infection in mice and/or cotton rats. To our knowledge, Novavax has produced the only current RSV VLP vaccine in clinical trials. In a phase 1, blinded, randomized, placebo-controlled clinical trial, the RSV-F VLP vaccine candidate elicited strong, dose-dependent RSV-F-specific and neutralizing antibody responses that were promoted in the presence of aluminum phosphate [100]. The vaccine appeared safe, immunogenic and reduced RSV infections.

## 7. Conclusions

Since the formalin-inactivated RSV vaccine failure in the 1960s, the majority of the effort has focused toward the development of live-attenuated RSV vaccines; however, a group of these candidates has failed due to under-attenuation, over-attenuation or failure to provide long-lasting immunity. In more recent years, numerous approaches to the development of RSV vaccines have been tested; among them, NPs, MPs and VLPs have become very popular, due to their easy formulation, stability, immunogenicity and safety. These particle-based vaccines have been shown to be highly protective in preclinical studies, and a few have progressed to clinical trials; it is expected that different vaccine formulations will be destined for different human target populations (i.e., newborns, kids, pregnant women and the elderly) with particle-based vaccine most likely being used for the adult and elderly population.
